# Insight Derived from Molecular Dynamics Simulations into Molecular Motions, Thermodynamics and Kinetics of HIV-1 gp120

**DOI:** 10.1371/journal.pone.0104714

**Published:** 2014-08-08

**Authors:** Peng Sang, Li-Quan Yang, Xing-Lai Ji, Yun-Xin Fu, Shu-Qun Liu

**Affiliations:** 1 Laboratory for Conservation and Utilization of Bio-Resources and Key Laboratory for Microbial Resources of the Ministry of Education, Yunnan University, Kunming, P.R. China; 2 College of Agriculture and Biological Science, Dali University, Dali, P.R. China; 3 Southwest Biological Diversity Laboratory, Kunming Branch of Chinese Academy of Sciences, Kunming, P.R. China; 4 Key Laboratory for Animal Genetic Diversity and Evolution of High Education in Yunnan Province, Kunming, Yunnan University, Kunming, P.R. China; 5 Human Genetics Center, School of Public Health, The University of Texas Health Science Center, Houston, Texas, United States of America; Russian Academy of Sciences, Institute for Biological Instrumentation, Russian Federation

## Abstract

Although the crystal structures of the HIV-1 gp120 core bound and pre-bound by CD4 are known, the details of dynamics involved in conformational equilibrium and transition in relation to gp120 function have remained elusive. The homology models of gp120 comprising the N- and C-termini and loops V3 and V4 in the CD4-bound and CD4-unbound states were built and subjected to molecular dynamics (MD) simulations to investigate the differences in dynamic properties and molecular motions between them. The results indicate that the CD4-bound gp120 adopted a more compact and stable conformation than the unbound form during simulations. For both the unbound and bound gp120, the large concerted motions derived from essential dynamics (ED) analyses can influence the size/shape of the ligand-binding channel/cavity of gp120 and, therefore, were related to its functional properties. The differences in motion direction between certain structural components of these two forms of gp120 were related to the conformational interconversion between them. The free energy calculations based on the metadynamics simulations reveal a more rugged and complex free energy landscape (FEL) for the unbound than for the bound gp120, implying that gp120 has a richer conformational diversity in the unbound form. The estimated free energy difference of ∼−6.0 kJ/mol between the global minimum free energy states of the unbound and bound gp120 indicates that gp120 can transform spontaneously from the unbound to bound states, revealing that the bound state represents a high-probability “ground state” for gp120 and explaining why the unbound state resists crystallization. Our results provide insight into the dynamics-and-function relationship of gp120, and facilitate understandings of the thermodynamics, kinetics and conformational control mechanism of HIV-1 gp120.

## Introduction

Acquired immune deficiency syndrome (AIDS), which is caused by a retro-virus termed human immunodeficiency virus (HIV), has been a life-threatening health problem and brought about catastrophic consequences to human society [1], [2]. The HIV type 1 (HIV-1) envelope is filled by many trimeric assemblies composed of exterior envelope glycoprotein gp120 and transmembrane glycoprotein gp41 [3], [4]. In contrast to the standard type of one-step fusion mechanism of enveloped viruses, the entry of HIV-1 into target cell needs two-step binding to two different receptors, the receptor CD4 and the co-receptor CCR5 or CXCR4 located on the surface of the host cell [5], [6], [7], [8]. Based on the evidences derived from biochemical and structural studies, people can now depict the general process of HIV-1 virus entry [9], [10], [11]. Initially, the binding of gp120 to the receptor CD4 leads to the exposure of its binding-site to the co-receptor CCR5 or CXCR4 [12], [13]; subsequently, the binding of gp120 to the co-receptor triggers its conformational rearrangements, which in turn facilitate the membrane fusion between the virus and host cell through insertion of the transmembrane protein gp41 into the cell membrane. This entry process involves a series of conformational rearrangements of gp120 and its multiple interactions with receptor and co-receptor, and therefore the dynamics of gp120 and its two-step binding to receptor and co-receptor are thought to play important roles in the virus infection and immune evasion [14].

The envelope glycoproteins gp120 and gp41 come from a common precursor, gp160, which is cleaved during transport into two components: the N-terminal receptor-binding component gp120 and the transmembrane component gp41 [3]. Three gp120 and three gp41 molecules associate on the virion surface through non-covalent interactions to form a trimetric complex, termed the viral spike, with gp120 and gp41 located in the exterior and interior of the spike, respectively [11]. Currently a number of HIV-1 gp120 core structures in complex with CD4 and other ligands (such as neutralizing antibodies 17b, b12 and N-terminus of the co-receptor CCR5) has been determined in a series of crystallographic studies [15], [16], [17], [18], [19]. These core structures are in the CD4-bound (or liganded) state and contain no the N- and C- termini and the loops V1/V2, V3 and/or V4. An unbound (or unliganded) gp120 core (i.e., in the state prior to binding to CD4, or in the non CD4-bound state) from the simian immunodeficiency virus (SIV) was also determined using the X-ray crystallographic technique [20]. Both the CD4-bound and CD4-unbound states demonstrate that the gp120 core is composed of the inner and outer domains, despite a very large conformational difference between these two states. In the CD4-bound state, two β-hairpins, namely the β5–β6 from the inner domain and the β21–β22 from the outer domain, come together to form a four-stranded “bridging sheet” minidomain, which is absent in the CD4-unbound state. The calculated C_α_ root mean square deviation (RMSD) between structural cores of the bound HIV-1 gp120 and the unbound SIV gp120 is ∼10 Å.

More recently, Kwon et al. resolved four crystal structures of the CD4-unbound gp120 cores that come from the HIV-1 clades B, C, and E [21]. These cores, when compared to the previously determined cores, contain the N-terminus and the extended V3 loop base (10 amino acid residues at the V3 base). However, they contain no the C-terminus and the V1/V2 loop. Therefore, the authors referred to these cores as the more-extended gp120 cores (core_e_) in order to distinguish them from the previously determined core_min_ [21]. It is surprising that these four unbound core_e_ structures resemble closely the CD4-bound state (the average C_α_ RMSD value between them is 1.4 Å) and differ substantially from the CD4-unbound SIV gp120 core_min_ (with average C_α_ RMSD of 9.7 Å to the SIV core_min_). However, thermodynamic studies of gp120 binding to CD4 [22] and a number of CD4-binding site antibodies [23], [24], [25] suggest that the unbound gp120 conformation should be substantially different from the CD4-bound conformation. To this end, these four CD4-unbound gp120 core_e_ structures, which are in the CD4-bound states, give rise to a challenge to the traditional view that achieving the CD4-bound state requires the ligand induction [21]. According to Kwon's crystallographic structures and the thermodynamic data on gp120-CD4 interaction [22], [26], [27], we infer that in solution the isolated gp120 molecules (the gp120 alone and not in the gp120_3_/gp41_3_ trimeric assembly) may exist in an ensemble of conformations comprising at least two distinct states, the unbound and CD4-bound states. Furthermore, based on the theory of FEL [28], [29], we speculate that the CD4-bound state of the unbound HIV-1 gp120 core_e_ should have a lower Gibbs free energy than the “true” unbound state (namely, the state similar to what has been observed in the CD4-unbound SIV gp120 core). This will lead to a larger population of the CD4-bound state than that of the unbound state in solution, making the former easier to be trapped in the crystallization condition [30]. On the contrary, the small population of the “true” unbound state will make it hard to trap this state in the crystallization condition. In addition, our previous simulation studies [31], [32], [33] demonstrate that the unbound gp120 displays larger conformational fluctuations and less stability than the CD4-bound gp120. These results, in conjunction with our above speculation, may explain why it is the CD4-bound state and not the “true” unbound state of gp120 that was resolved in the crystallographic study [21].

Although the different conformational states of the static crystallographic snapshots provide invaluable insight into the function of gp120, detailed information on many dynamic aspects of gp120, including the dynamic features, molecular motions, conformational transition, conformational diversity, gp120-ligand binding, and how the dynamics are regulated, have remained unclear when only static structures are available. In addition, the currently available gp120 structures are all the structural cores that lack certain external loops and/or the N- and C-termini. The loops, due to their high flexibility and large conformational entropy, generally have fundamental role in determining/modulating the dynamic behaviors of proteins such as the large concerted motions, conformational transition, and protein-ligand recognition and binding [30], [34], [35], [36]. In the case of gp120, it has been assumed that its external loops play an important role in regulating dynamics of the protein, thus determining the thermodynamic (i.e., the relative populations of the conformational states) and kinetic (i.e., conformational conversion between these states) properties of gp120 [21], [30] and, ultimately, its functions — the ligand binding and viral immune evasion.

In this paper, two near-full-length three-dimensional (3D) structural models (containing the N-, C-termini and the loops V3 and V4) of gp120 in the CD4-unbound and CD4-bound conformational states were built, respectively, using the comparative modeling method. These two models were subsequently used as starting structures for standard MD and metadynamics simulations [37], [38]. The aims of this paper are to compare the differences in dynamics and molecular motions between these two states, and to verify our above speculation about the equilibrium conformational distribution of these two states. Results in this study will aid in a better understanding of the thermodynamics and kinetics of HIV-1 gp120 and further, of its functional properties.

## Materials and Methods

### Sequence preparation

The amino acid residue sequence of the HIV-1 JR-FL isolate gp160 precursor (accession number Q75760) [16] was obtained from SwissProt protein sequence database [39]. The sequence segments for the signal peptide (residues 1–31) and transmembrane glycoprotein gp41 (residues 493–847) were removed and the segment for the V1/V2 loop (residues 127–191) was replaced by GAG. This leads to a final primary target sequence of gp120 that consists of 401 residues, which will be used for building the structural models using the comparative modeling method.

### Preparation of template structures

Template structures of the gp120 core were obtained from PDB protein structure database [40]. PDB entries 3JWD [19] and 2B4C [16] contain crystal structures of HIV-1 gp120 core in the CD4-bound state. In addition to the core structure, the chain A of 3JWD contains the N- and C-termini but no the V3 loop, and the chain G of 2B4C contains the V3 loop but no the N- and C-termini. Therefore, both 3JWD chain A and 2B4C chain G were used as templates to construct an as complete as possible gp120 model in the CD4-bound state. The sequence identities between our target sequence and structure templates 2B4C and 3JWD are 99% and 80%, respectively.

PDB entry 3FUS [20], which contains neither the V3 loop nor the N-, C-termini, is a crystal structure of the SIV gp120 core in the CD4-unbound state. It is worth noting that 3FUS is the only currently available experimental structure of gp120 in the unbound state. The sequence identity and similarity between the SIV gp120 and our target sequence are 35% and 70%, respectively. Therefore, it is reasonable to use 3FUS as the template for building the HIV-1 gp120 model in the unbound state. For the all above templates, the sugar groups and crystal water molecules were removed and only the gp120 atomic coordinates were retained for subsequent modeling.

### Structural model construction

**Figure 1:**
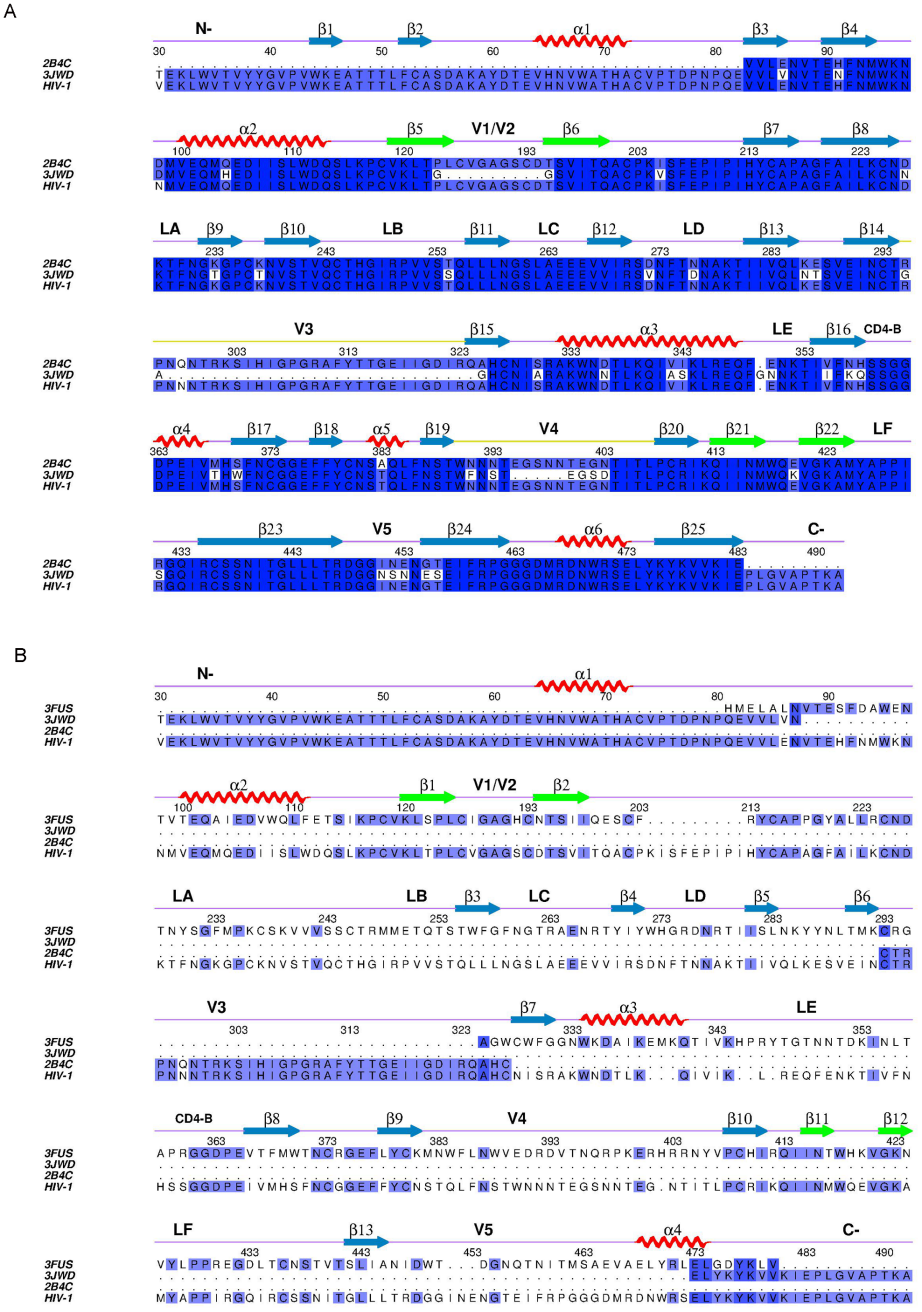
Multiple sequence alignments between gp120 target sequence and sequences of the selected structural templates. (A) and (B) are alignments for constructing gp120 structural models in the CD4-bound and CD4-unbound states, respectively. HIV-1 represents the target sequence coming from HIV-1 JR-FL isolate gp160 precursor with Swiss-Prot accession number Q75760. 3JWD, 2B4C, and 3FUS represent sequences of crystal structures with PDB entries 3JWD (chain A), 2B4C (chain G), and 3FUS (chain A), respectively. It should be noted that in (B) only the segments of the N-, C-termini from 3JWD (chain A) and of the loop V3 from 2B4C (chain G) were used as templates for building corresponding structural parts of gp120. Strongly and weakly conserved residues were shaded in dark and light blue, respectively. The secondary structural elements were assigned according to the templates with red spirals and blue arrows representing α helices and β strands, respectively. The green arrows represent β strands (i.e., β5–β6 and β21–β22 in (A), and β1–β2 and β11–β12 in (B)) that can participate in the formation of the bridging sheet. The “GAG” sequence in the V1/V2 loop of gp120 is the consequence of truncation.

The MODELLER software package [41] was used to build the gp120 structural models. In order to obtain an as complete as possible model in the bound state, we aligned simultaneously the target sequence to the chain G of 2B4C and chain A of 3JWD after a structural alignment between these two templates (Figure 1A). Analogously, structural alignment between 3FUS, the structural segments for the V3 loop from 2B4C and for the N- and C-termini from 3JWD was performed. This was followed by aligning the target sequence to these templates (Figure 1B) in order to obtain an as complete as possible structural model in the unbound state. 20 structural models were generated for the bound and unbound gp120, respectively, and the molecular dynamics simulated annealing (SA-MD) was performed to refine these models. The structural assessment was performed using PROCHECK [42]. The models having the minimum value of molecular probability density function were selected for following MD simulations.

### MD simulations

Energy minimizations and MD simulations were performed using GROMACS package [43] with the GROMOS96 43a1 force field. Before MD simulation, the unreasonable atomic contacts and stereochemical conflicts within the initial structures were removed by the steepest descent following the conjugate gradient energy minimizations in vacuum. The energy-minimized structures were solvated using the single point charge (SPC) water molecules [44] in cubic boxes with the minimum solute-box wall distances of 11 and 10 Å, resulting in a total of 219,304 and 219,283 atoms in the protein-solvent systems of the CD4-unbound and CD4-bound gp120, respectively. The different minimum solute-box wall distances were set in order to give similar numbers of atoms in each simulation system. In order to perform simulation at 100 mM NaCl while maintaining system's electroneutrality, 137 Na^+^ and 136 Cl^−^ were introduced by replacing water molecules in the unbound gp120-solvent system. For the bound gp120-solvent system, 138 Na^+^ and 138 Cl^−^ were introduced. Before the production MD runs, 100-ps position-restrained MD simulations were performed to make good contacts between the solute and solvent. In order to improve the conformational sampling, 6 independent 15-ns production MD simulations were performed for each system with different initial atomic velocities assigned. The obtained MD trajectories for the same system with different initial velocities are referred to as replica 1 to replica 6.

The following MD protocols were used: the integration time step was 2 fs; center-of-mass motion was removed every time step; non-bonded pairs were updated every 10 time steps; electrostatic interactions were treated with Partial-Mesh Ewald (PME) summation algorithm [45] with interpolation order of 4, Fourier grid spacing of 0.135 nm and Coulomb radius of 1.0 nm; van der Waals (VDW) interaction cut-off radius was 1.4 nm; protein and non-protein (solvent and ions) components were independently coupled to a 300 K heat bath with a coupling constant τ_t of 0.1 ps; and the pressure was maintained by weakly coupling the system to an external pressure bath at 1 atm with a coupling constant τ_p of 0.5 ps [46]; initial atomic velocities were taken at startup from a Maxwell distribution corresponding to a temperature of 300 K; LINCS algorithm [47] with order 4 was used to constrain the bond lengths to their equilibrium positions; structural coordinates were saved every 10 ps.

### Evaluations of trajectory equilibration and sampling convergence

In order to evaluate the equilibration of MD trajectories, the time-dependent backbone RMSD values relative to the starting structures were calculated. The results (Figure S1) show that for each replica of both simulation systems, ∼5 ns is required to reach relatively stable RMSD values. Subsequently, the equilibrium regions (5–15 ns) of the replicas for each simulation system were concatenated to obtain a single 60-ns joined trajectory, which represents different sampling directions around the starting structure.

The cosine content of the first few eigenvectors derived from ED analysis of MD trajectory (See below for details of the ED analyses), which is a measure for similarity to random diffusion, is a good indicator for sampling convergence. This value ranges between 0, no cosine, and 1, a perfect cosine. It has been shown that when the cosine content of the first few eigenvectors is close to 1, the large-scale motions along the eigenvectors resemble random diffusion, representative of insufficient sampling [48], [49].For both the unbound and bound gp120 simulation systems, the cosine content values of the first 4 eigenvectors for the 6 individual replicas and the single joined trajectories were computed and compared. The results (Table S1) show that the cosine values of the independent replicas are generally larger than those of the corresponding joined trajectories; particularly for the first two eigenvectors the values of the joined trajectories are one to two orders of magnitude smaller than the corresponding values of the replicas. These results indicate that the replicas partly describe the random diffusion motions while the joined trajectories sample sufficiently large conformational space. Interestingly, the relatively larger values of the joined unbound gp120 trajectory than those of the joined bound gp120 trajectory suggest that the former may experience relatively larger random diffusion motions, harder to reach sampling convergence than the latter during MD simulations.

To this end, for both the unbound and bound gp120, their single 60-ns joined trajectories were used for subsequent geometric property and ED analyses, and the first two eigenvectors extracted from the joined trajectories were used for metadynamics simulations and FEL reconstructions except where noted.

### Geometric property calculations

The geometrical properties of the unbound and bound gp120 structures during MD simulations, such as the number of hydrogen bonds (NHB), solvent accessible surface area (SASA), number of native contacts (NNC), radius of gyration (Rg), and number of residues in SSEs, were calculated using the programs g_hbond, g_sas, g_mindist, g_gyrate, and do_dssp [50] within GROMACS, respectively.

### ED analyses

Essential dynamics (ED) method [51], [52] or, equivalently, the principal component analysis (PCA) in mathematics, is a powerful tool for filtering large-scale concerted motions from a structural ensemble or MD trajectory. After diagonalization of the covariance matrix built from the atomic fluctuations in a trajectory, a set of eigenvectors and corresponding eigenvalues can be obtained. The eigenvectors are the directions in conformational space and represent the collective motions of atoms along those directions. The eigenvalues represent the mean square fluctuations (MSF) of atoms along the corresponding eigenvectors. It has been shown that the first few eigenvectors define an essential conformational subspace within which the most significant large-scale concerted motions (or the major motion modes) take place [53]. For the two simulation systems, the C_α_ atom covariance matrices were built and diagonalized using the program g_covar within GROMACS package. The projections of trajectories onto the eigenvectors were performed using the g_anaeig program within GROMACS. In order to visualize vividly the large concerted motions along the eigenvectors, a Tcl script [54] combined with VMD program [55] was used to plot porcupine representations of these motions. For instance to visualize the motions along eigenvector 1, a cone is drawn for each residue starting from the C_α_, projecting in the direction of component of the first eigenvector with the length of the cone representing the fluctuation amplitude.

Combined ED is a useful method for comparing ED properties of two simulations on similar systems [56]. In this study, the combined ED analysis was performed on a trajectory constructed through concatenating the unbound and bound gp120 MD trajectories. Analysis and comparison of the properties (i.e., the projection distributions/average values and mean square displacements (MSD)) of different parts of a projection along the combined eigenvector provide a powerful way for accessing the similarities or differences in equilibrium fluctuations (or equilibrium states) and dynamics between these two forms of gp120.

### Metadynamics simulations

In order to reconstruct the FEL of the gp120 molecule, well-tempered metadynamics simulations [57] were performed on our unbound and bound gp120 models. The metadynamics algorithm can accelerate rare event sampling and help the system escape from free energy minima through adding an external history-dependent potential acting on few properly chosen degrees of freedom, termed also as collective variables (CV) [37]. Here we used a newly developed variant of metadynamics, named the well-tempered metadynamics, in which the variations on time of the bias potential are decreased with a specific law that allows to avoid exploration of the unphysically high free energy regions, thus guaranteeing a smooth convergence of the simulation [57]. It has been shown that coupling well-tempered metadynamics with a set of CVs from ED method provides an efficient reconstruction of the FEL of a protein [58], [59]. In this study, we chose projections of trajectory onto the eigenvectors 1 (PC1) and 2 (PC2) as the CV1 and CV2, respectively, in the metadynamics simulations.

The principal parameters used in the well-tempered metadynamics simulations are: the initial Gaussian height was 0.4 kJ/mol and added every 2 ps; the Gaussian width was 0.35 nm; the bias factor was set to 10. Other simulation parameters and conditions were the same as those in the standard MD simulations. The initial structures for metadynamics simulations were the final snapshots of the joined standard MD trajectories. The well-tempered metadynamics simulations were run for 100 ns for both the unbound and bound gp120 models using GROMACS augmented with Plumed [60]. The FELs were constructed using weighted histogram analysis method [61] implemented in Plumed [60].

## Results

### Structural and geometric comparisons between the unbound and bound gp120 models

**Figure 2:**
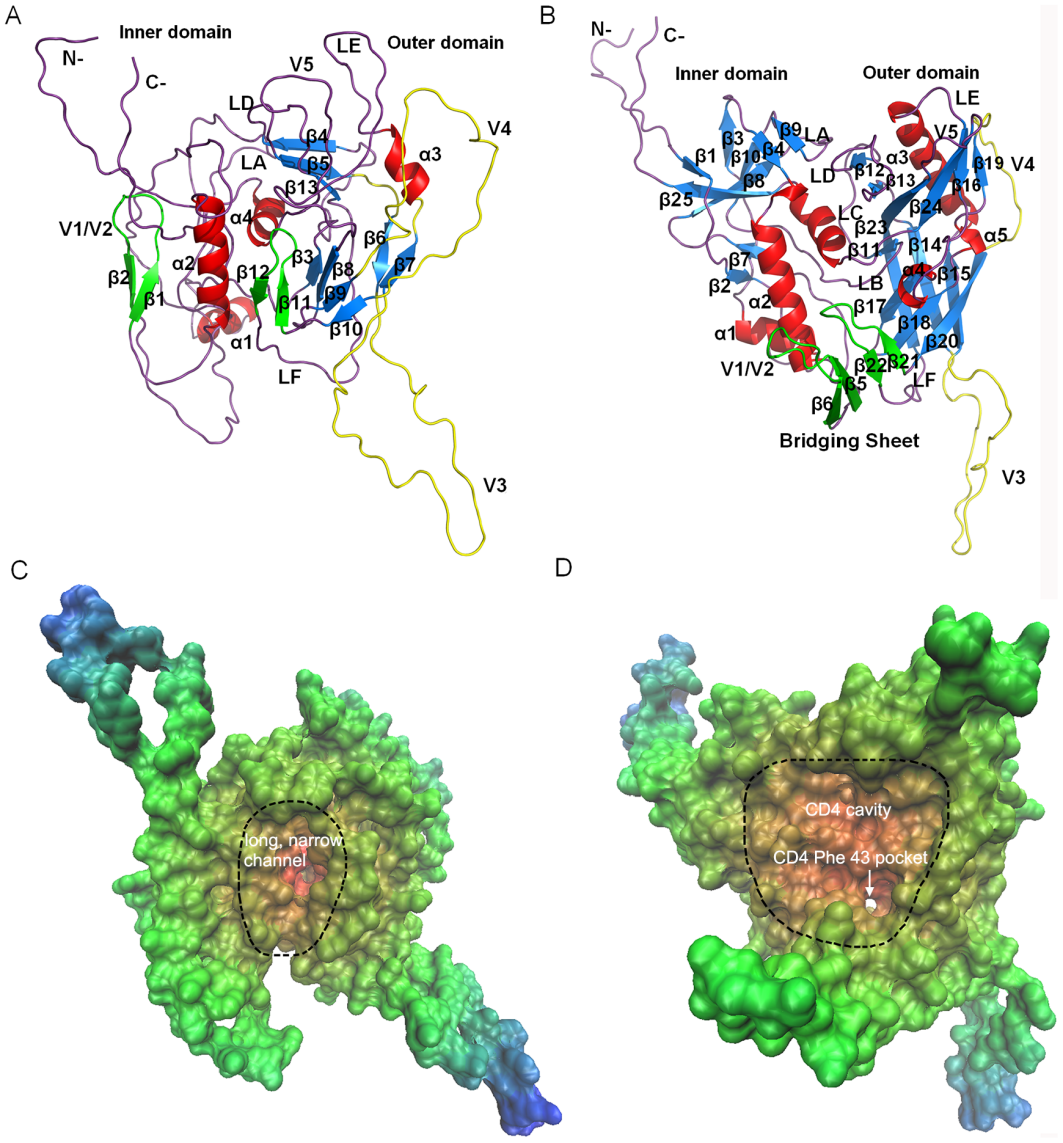
Structures and molecular surfaces of gp120 homology models. (A) and (B) are ribbon representations of the unbound and bound gp120 models, respectively. α helices are in red, β strands in blue (except for those that are able to participate in the formation of the bridging sheet), loops V3 and V4 in yellow, and bridging sheet (only in the bound gp120 models) in green. (A) and (B) were generated using the Pymol program [69]. (C) and (D) are solvent accessible surfaces of the unbound and bound gp120 models, respectively. Solvent accessible surfaces were colored according to the accessibility of residues to solvent, ranging from blue (most accessible) to red (least accessible). The long, narrow channel in the unbound gp120, and the large CD4 cavity and CD4 Phe 43 pocket in the bound gp120 were circumscribed by black dashes. These two plots were generated using the VMD program [55].

The ribbon representations of the gp120 structural models in the CD4-unbound and CD4-bound states were shown in Figures 2A and B, respectively. Both models consist of two major domains, the inner and outer domains. As shown in Figure 2A, the inner domain of the unbound model consists of the N-, C-termini, a two-stranded antiparallel β-hairpin (β1–β2), a two-helical bundle (α1–α2), and the helix α4 (which is located between the inner and outer domains). The main body of the outer domain of the unbound model is a stacked antiparallel β-barrel that lies alongside the inner domain. The V3 loop, which acts as a connection between the β6 and β7, lies beneath the distal end of the outer domain. The V4 loop protrudes from the right side of the outer domain and adopts an open conformation. In the case of the bound gp120 (Figure 2B), its inner domain comprises a six-stranded β-sandwich at the termini-proximal end in addition to the two-helical bundle (α1–α2) and α6 (corresponding to the α4 in the unbound model). Apparently, its outer domain possesses more and longer SSEs than that of the unbound gp120, although both outer domains share a common structural organization. Like what has been observed in the unbound gp120 model, the modeled loops V3 and V4 in the bound model also protrude away from the surface of the outer domain and display random-coiled conformation. It is important to note that, in the bound model, the hairpin β5–β6 (also called V1/V2 stem) from the inner domain and the hairpin β21–β22 from the outer domain constitute an antiparallel four-stranded bridging sheet minidomain that stands below the distal ends of both domains. However, such a minidomain is not found in the unbound form because of a long separation distance (∼23 Å) between the corresponding hairpins β1–β2 and β11–β12. In the bound form, the hairpin β21–β22, V1/V2 stem, loops LD, LE and V5, and α4-β17 and β23-α5 constitute an unusually large CD4-binding cavity, at whose bottom the CD4 Phe43 binding pocket lies (Figure 2D). The unbound form has no such a cavity, but instead contains a long, narrow channel that is composed of the α2, α4, β11–β12, and CD4-binding loop (CD4-BL) at the intersection surfaces of the inner and outer domains (Figure 2C).

In addition to the static structural comparison, we further calculated the average geometric properties including NHB, SASA, NNC, Rg and SSE based on the single joined trajectories to compare quantitatively the structural feature and stability between these two gp120 models. Table 1 shows that the bound gp120 has more NHB, NNC, and SSE residues but smaller SASA than the unbound gp120, indicating more numbers of inter-atomic interactions/contacts and regular structural elements, and a more compact packing in the bound gp120 than in the unbound form. In addition, for all the geometric properties, the unbound gp120 has larger standard deviations than the bound gp120, demonstrating that the former experienced larger structural variations during simulations. These results, together with a lower potential energy of the bound gp120, indicate that during MD simulations the bound form was on average in a more compact and stable state than the unbound gp120, in agreement with the static structural comparison.

**Table 1:** Average geometrical properties (standard deviations are shown in parentheses) calculated from the equilibrium MD trajectories of the unbound and bound gp120 models.

	NHB^a^	SASA^b^ (Å^2^)	NNC^c^	Rg^d^ (Å)	SSE^e^			ENE^f^ (kJ/mol)
					α-helix	β-sheet	Turn	
unbound	233.5 (10.9)	20568 (709)	195269 (2787)	22.3 (0.9)	29 (7)	87 (10)	27 (8)	−3.13×10^6^ (1.84×10^3^)
bound	265.9 (10.6)	20040 (317)	196183 (1318)	23.5 (0.4)	53 (4)	142 (10)	22 (5)	−3.14×10^6^ (1.81×10^3^)

a Number of hydrogen bonds. A hydrogen bond is considered to exist when the donor-hydrogen-acceptor angle is larger than 120° and the donor-acceptor distance is less than 3.5 Å.

b Total solvent accessible surface area.

c Number of native contacts. A native contact is considered to exist if the distance between two atoms is less than 6 Å.

d Radius of gyration.

e Number of residues in the corresponding secondary structure elements.

f Potential energy calculated with GROMOS96 43a1 force field using PME method.

Taken together, both the static and dynamic structural/geometric comparisons between the two gp120 models reveal that the bound form has more numbers of inter-atomic contacts and SSEs, longer SSEs and shorter linkers that separate SSEs, a more compact packing, and a more stable structure than the unbound form. It should be mentioned that backbone RMSD between these two models is 14.4 Å, indicating a distinctly different structural organization or inter-domain arrangement between the unbound and bound gp120. We conclude that gp120 is a peculiar protein that differs from the ordinary globular proteins and exhibits a large conformational heterogeneity under different functional states.

### Structural flexibility

**Figure 3:**
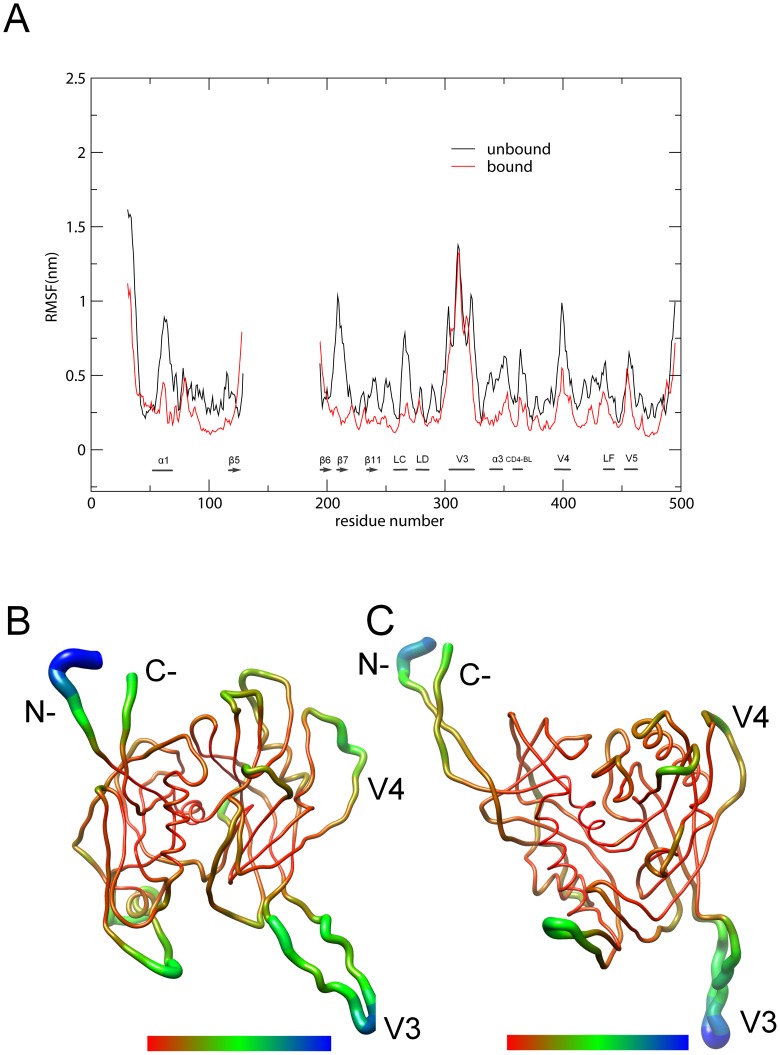
Comparison between the structural flexibility of the unbound and bound gp120 models. (A) Per-residue average backbone RMSF profiles calculated from MD trajectories of the unbound (black line) and bound (red line) gp120. Note that the residues 127–191 corresponding to the V1/V2 loop are absent in our models. Loops V3, V4, V5, LC, LD and LF, and some of SSEs were marked according to the bound gp120 model. CD4-BL represents the CD4-binding loop. (B) and (C) are 3D backbone representations of the unbound and bound gp120 models that are colored according to the per-residue average backbone RMSF values, respectively. The color scale ranges from red to blue, with red corresponding to the thinnest backbone with the lowest RMSF value and blue corresponding to the thickest backbone with the highest RMSF value. (B) and (C) were generated using UCSF Chimera [70].

Per-residue average backbone root mean square fluctuation (RMSF) values were computed based on the MD trajectories to evaluate and compare the structural flexibility between the unbound and bound gp120. Figure 3 shows the RMSF values as a function of residue number as well as the 3D backbone representations of gp120 colored according to the RMSF values. Figure 3A shows clearly that the bound gp120 has an overall lower flexibility (or higher rigidity) than the unbound gp120 with the exception of only a very limited number of sites such as the N-terminus and hairpin β5–β6. Also of note is that the V3 loops of both models have similar RMSF values. For both forms of gp120, the common high-flexibility regions, arbitrarily defined as those with RMSF>0.3 nm, include the loops V3, V4 and V5, and the N- and C-termini (Figure 3A). The structural cores of both forms of gp120 have lower RMSF values than the external loops and, as thus demonstrate a high rigidity, which can be more intuitively observed in Figures 3B and C. The high rigidity of the structural cores is due to their small structural deviations with respect to the starting structures during MD simulations; while the increased structural deviations or instability of the entire structures arise mainly from the high flexibility of the external loops (Table S2). We consider that it is the high flexibility of the external loops that makes it difficult to crystallize gp120 unless they are truncated.

### ED analyses

**Figure 4:**
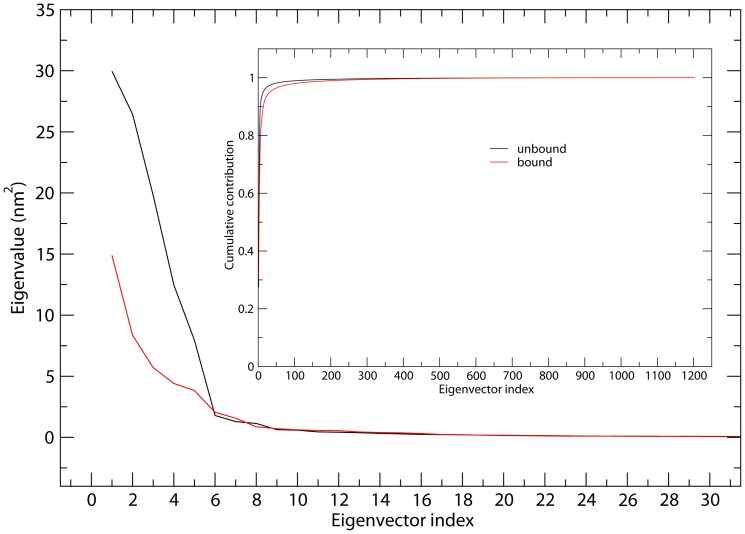
Eigenvalues for the unbound and bound gp120 models as a function of eigenvector. The main plot shows the eigenvalues of only the first 30 eigenvectors. The inset shows the cumulative contribution of all the 1203 eigenvectors to the total MSF. Unbound: black line; bound: red line.

ED analyses of MD trajectories of the unbound and bound gp120 models reveal that only a few eigenvectors possess significant eigenvalues (Figure 4). Diagonalization of the covariance matrices obtained the total MSF values of 109.0 and 50.0 nm^2^ for the unbound and bound gp120, respectively, indicating that the unbound gp120 experienced larger fluctuation amplitude during simulations. In particular, it is apparent that each of the first 5 eigenvectors has a larger eigenvalue for the unbound than for the bound gp120 (Figure 4), reflecting larger collective atomic fluctuations of the unbound gp120 along these eigenvectors. In addition, for the unbound and bound gp120, the first 4 and 10 eigenvectors contribute 81.4% and 93.7% and, 66.8% and 86.1% to the total MSF (see Figure 4, inset), respectively. Therefore, the first 10 eigenvectors, especially the first 4 eigenvectors, can be considered to span an essential subspace within which the large-scale concerted motions take place.

**Figure 5:**
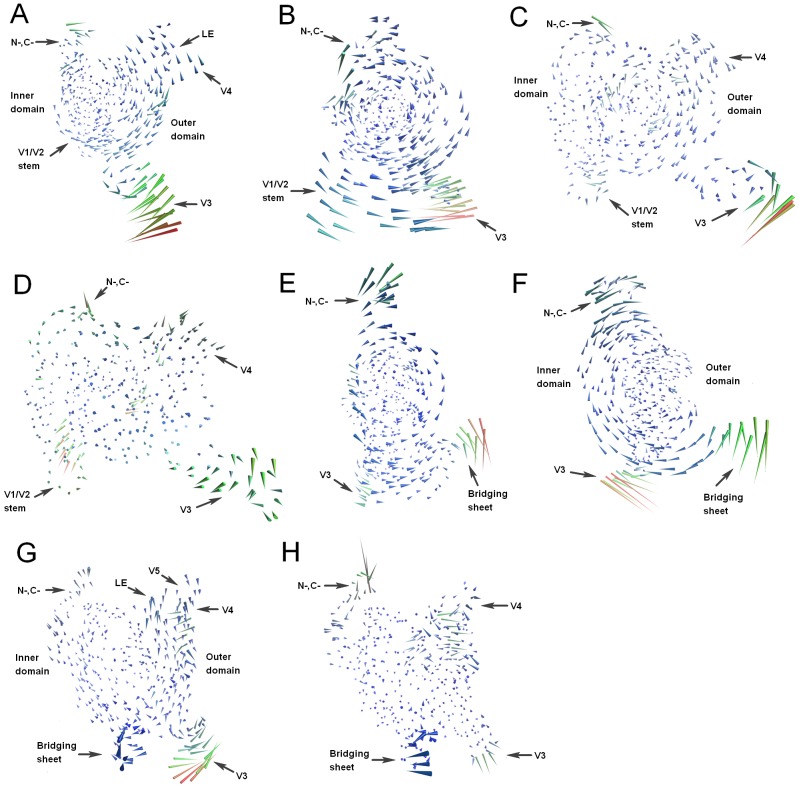
Porcupine plots of the large concerted motions along the first 4 eigenvectors. (A)–(D) are motion modes along eigenvectors 1–4 of the unbound gp120, respectively. (E)–(H) are motion modes along eigenvectors 1–4 of the bound gp120, respectively. For (A), (C), (D), (G), and (H), the view is looking towards the center between the inner and outer domains. For (B), (E), and (F), the view is from the inner to outer domains. The color of the cone/porcupine ranges from blue to red, with blue corresponding to the shortest cone/porcupine with the smallest atomic displacement magnitude and red corresponding to the longest cone/porcupine with the largest atomic displacement magnitude.

Figure 5 shows, in porcupine representation, the large concerted motions (or motion modes) along the first 4 eigenvectors of the unbound and bound gp120 models. The most significant motion mode along eigenvector 1 of the unbound gp120 can be described as common rotations of the inner and outer domains around an axis running through the center between these two domains, resulting in a large anticlockwise vortex as shown in Figure 5A. The V3 loop from the outer domain and the N- and C-termini from the inner domain, which have the largest conformational displacements, rotate concertedly in an opposite direction relative to the large vortex. Regions with a moderate displacement magnitude include the V1/V2 stem of the inner domain and the loops V4, V5 and LE of the outer domain. The smallest displacements were observed in the major part of the structural core. The motion mode along the second eigenvector of the unbound gp120 displays as a large anticlockwise vortex that rotates around an axis connecting the centers of the inner and outer domains (Figure 5B). The largest displacements occur on the V3 loop and V1/V2 stem, which move in opposite directions with respect to each other, leading to a mutual approach between them. Other external loops have moderate displacements and rotate concertedly around the structural core, which has the smallest displacement magnitude. In the third ranked mode of the unbound gp120 (Figure 5C), the inner and outer domains rotate in opposite directions around an axis running through the center between these two domains, resulting in a twist of one domain relative to the other. The entire structure has a relatively small displacement magnitude except for the distal end of the V3 loop, which moves in an opposite direction with respect to its base, resulting in a twist of the this loop. It appears that in the fourth ranked mode, there is no apparent rotation or twist between the inner and outer domains (Figure 5D). However, the opposite motion directions between the inner and outer domains, in particular between their upper parts, seem to widen the gp120 molecule.

In the case of the bound gp120, its first ranked motion mode (Figure 5E) resembles the second ranked mode of the unbound gp120, both displaying as a large anticlockwise vortex resulting from common rotations of the major parts of the inner and outer domains. The largest structural displacements occur on the N- and C-termini, V3 loop, and bridging sheet, with the N- and C-termini rotating in the same direction as the vortex while the latter two regions in an opposite direction relative to the vortex. In the second ranked mode of the bound gp120 (Figure 5F), the inner domain rotates clockwise while the outer domain rotates anticlockwise, resulting in a twisting motion between these two domains. Like what has been observed in the first mode, the N- and C-termini, V3 loop, and bridging sheet also have the largest displacements. However, the V3 loop and bridging sheet move in opposite directions relative to each other. The third ranked mode of the bound gp120 (Figure 5G) displays as a less obvious clockwise vortex resulting from common weak rotations of the inner domain and most part of the outer domain around an axis running through the center between these two domains. The outer domain, especially the loops LE, V4, and V5, has a relatively larger displacement magnitude than the inner domain. The V3 loop has the largest displacement magnitude and moves in an opposite direction relative to the vortex. The bridging sheet has a moderate displacement magnitude and experiences a twisting motion. The fourth ranked mode of the bound gp120 exhibits no apparent rotational/twisting motion between the inner and outer domains (Figure 5H). The largest displacements occur on the N- and C-termini, V3 loop, and upper part of the outer domain, whose motions seem to narrow/thin to a certain extent the gp120 structure. Also of note is the bridging sheet, which has a moderate displacement magnitude and moves away from the V3 loop.

**Figure 6:**
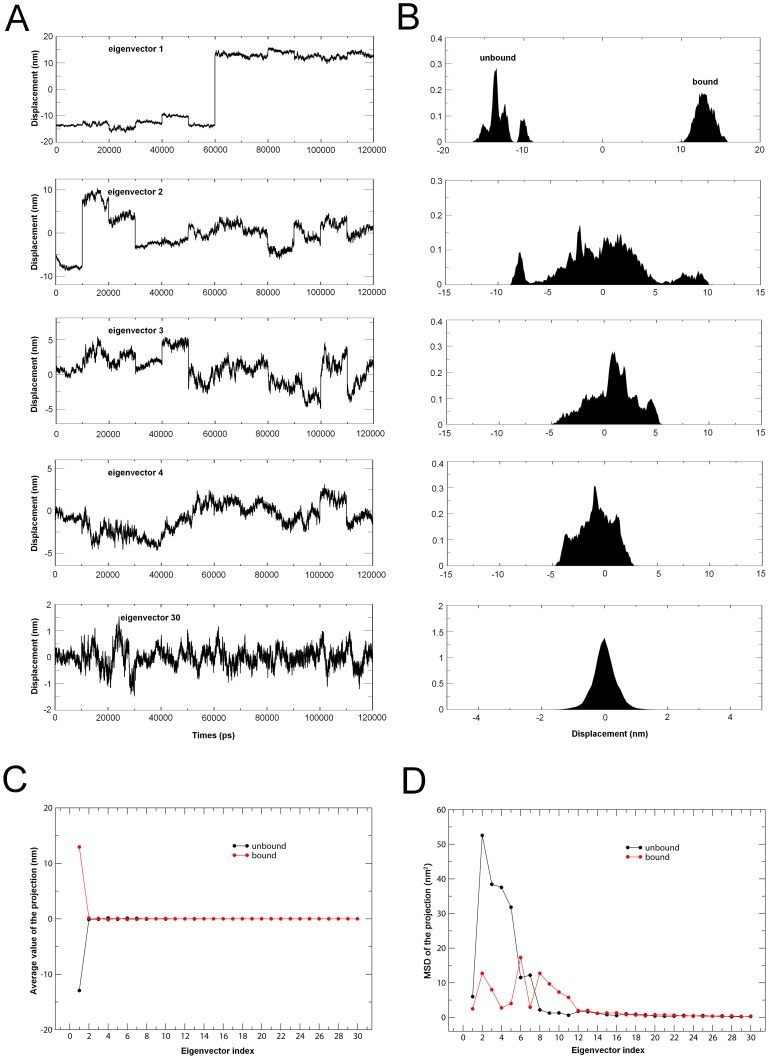
Properties of the projections of the merged trajectory onto the combined eigenvectors. (A) Projections of the merged trajectory (unbound: 0–60 ns; bound: 60–120 ns) onto the combined eigenvectors of 1–4 and 30. (B) Distributions of the corresponding eigenvector projections. Distinctly different distribution can only be found in the projection along the eigenvector 1. (C) Average values of the projections of the first 30 eigenvectors as a function of eigenvector index. (D) MSD values of the projections of the first 30 eigenvectors as a function of eigenvector index. The average and MSD values of the projection along a combined eigenvector were calculated separately for the two equal halves of the projection that correspond to the unbound and bound parts of gp120, respectively.

Combined ED analysis was performed to compare the ED properties between the unbound and bound gp120 models. Figure 6 shows the projections of the merged trajectories onto the combined eigenvectors as well as the properties of these projections. As shown in Figures 6A, B and C, only in the case of the first eigenvector can the projection be found to have significantly different distributions and average values, indicating distinctly different large concerted motions or equilibrium states between these two forms of gp120 along this combined eigenvector. Also worth noting is that the projection of the unbound gp120 exhibits a relatively inhomogeneous distribution (e.g., four peaks) while that of the bound gp120 exhibits a normal distribution (Figure 6B: top panel), suggesting a larger conformational heterogeneity (or more conformational substates) of the unbound gp120 along the first combined eigenvector.

The projection distributions of the second to fourth combined eigenvectors demonstrate a gradually increasing degree of overlap between the two equal halves of the projections, and particularly that of the eigenvector 30 exhibits a homogeneous normal distribution (Figure 6B: bottom panel). These results indicate that the similarity between the collective motions of these two forms of gp120 is gradually increasing with increased eigenvector index. This trend can be clearly observed in Figure 6C, which shows the comparison between the average values of the eigenvector projections of the unbound and bound gp120. Figure 6D shows the comparison between the MSD values of the eigenvector projections, which provide information about the difference in dynamics/flexibility between the unbound and bound gp120. A common feature for both forms of gp120 is that the projections of their first few eigenvectors have relative larger MSD values than the other eigenvector projections, suggesting that both forms of gp120 experience larger conformational shifts/changes within the subspace spanned by the first few eigenvectors. The most significant conformational shifts occur along the eigenvectors 2 and 6 for the unbound and bound gp120, respectively. Of particular note is that for the unbound gp120, its first five eigenvector projections have significantly higher MSD values than the corresponding values of the bound gp120. This indicates that the unbound gp120 experiences larger conformational shifts (and hence has higher flexibility) than the bound gp120 in the essential subspace of the first five eigenvectors, in agreement with the results of structural and geometric analyses.

### Free energy calculations

**Figure 7:**
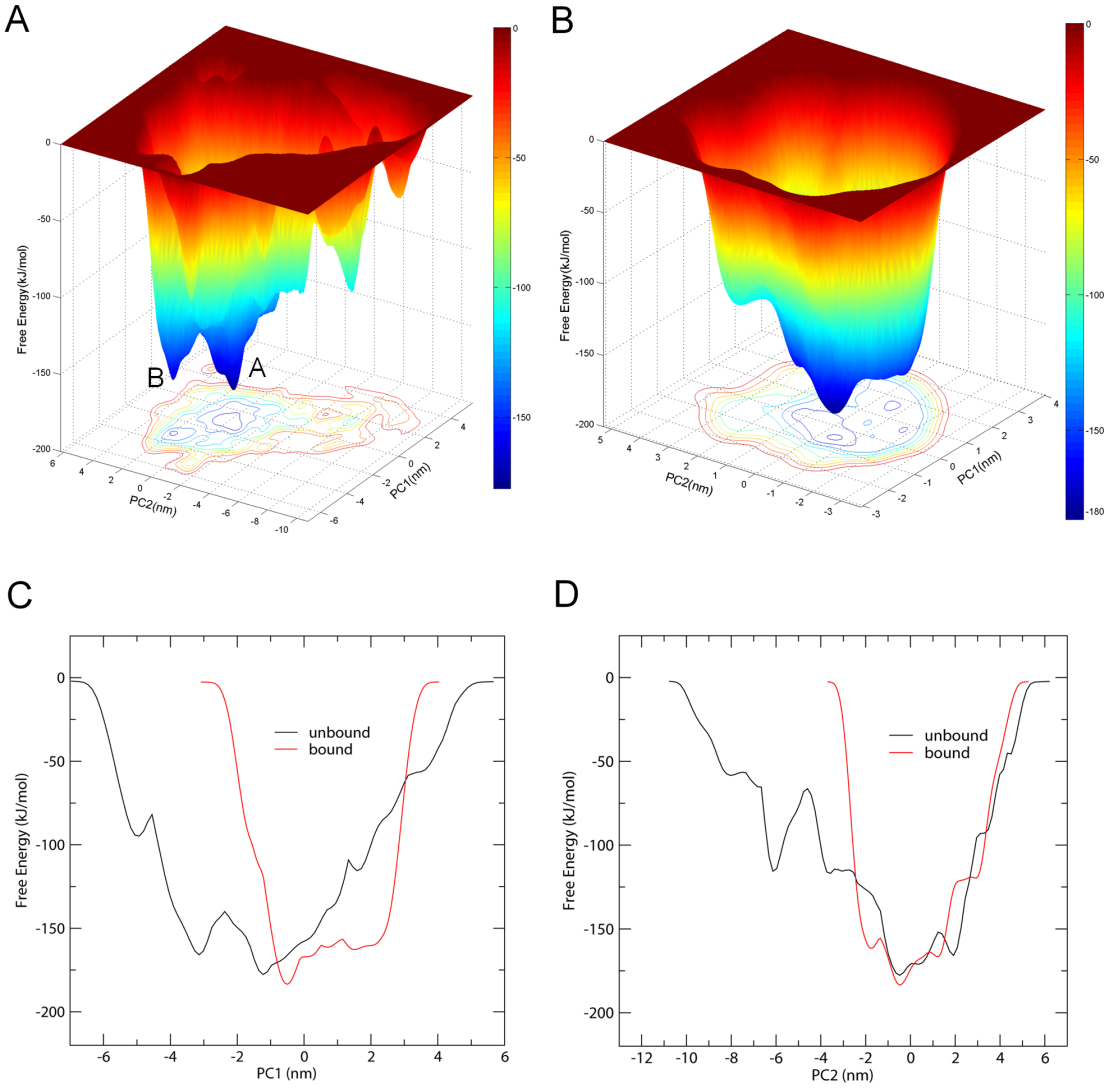
Constructed FELs and free energy profiles for the unbound and bound gp120 models. (A) and (B) are FELs for the unbound and bound gp120 as a function of projections of the MD trajectory onto the first (PC1) and second (PC2) eigenvectors, respectively. The color bar represents the free energy value in unit of kJ/mol. (C) and (D) are 1D free energy profiles of these two forms of gp120 (unbound: black line; bound: red line) as a function of PC1 and PC2, respectively.

The FELs were constructed by performing metadynamics simulations with projections of the first (PC1) and second (PC2) eigenvectors as the CV1 and CV2, respectively. These two eigenvectors span an essential subspace that contributes ∼50% to the total MSF of the conformation space sampled by the standard MD simulations. Figures 7A and B show the constructed FELs for the unbound and bound gp120, respectively, both of which present a funnel-like shape. For the unbound gp120, there are two main free energy wells/basins in the global free energy minimum region (which is arbitrarily defined as that with free energy <−163.0 kJ/mol) of the landscape, suggesting two main conformational substates, namely A and B residing within these two wells, respectively. The free energy well A is slightly larger and deeper than the well B, indicating that the substate A has a relatively larger population and lower free energy than the substate B. In the case of the bound gp120, there is only one free energy well in the global free energy minimum region, indicating only one stable conformational state residing within this well. A comparison between the full views of the FELs for these two forms of gp120 reveals that the FEL of the unbound gp120 spans larger ranges of PC1 and PC2 and exhibits a more rugged free energy surface than that of the bound gp120. For instance, the FEL of the unbound gp120 spans ranges of ∼11.6 and ∼16.0 nm along the PC1 and PC2, respectively, while the corresponding ranges for the bound gp120 are ∼7.1 and ∼7.6 nm, respectively. These results indicate that the unbound gp120 sampled a larger free energy surface than the bound gp120 during simulations. Furthermore, the unbound gp120 FEL contains more number of local free energy minima either in the global free energy minimum region (i.e., the funnel bottom) or in the region outside the global free minimum (i.e., the funnel wall), resulting in a more rugged and complex FEL of the unbound gp120 compared to that of the bound gp120.

In order to more visually compare the difference in characteristics between the FELs of these two forms of gp120, we further calculated the 1D free energy profile as a function of PC1 (Figure 7C) and of PC2 (Figure 7D). Apparently, along either the PC1 or the PC2, two global free energy minimum wells were observed for the unbound gp120 whereas only one for the bound gp120. It is important to note that the bound gp120 has a lower minimum free energy value than the unbound gp120, with an estimated difference of ∼−6.0 kJ/mol. In other words, the bound state residing within the global free energy minimum well has a lower free energy of ∼6.0 kJ/mol than the substate A of the unbound gp120, which resides within the free energy well that has the absolute minimum free energy value. In addition, for the unbound gp120, both of its PC1 and PC2 profiles are wider and rougher than the corresponding profiles of the bound gp120, in agreement with above analyses of the 2D FELs.

It should be pointed out that the FELs and free energy profiles constructed from our metadynamics simulations are incomplete and represent a major portion of the native landscape due to the limited conformational sampling and large dimensionality reduction, and as thus the transition between the unbound and bound gp120 states cannot be observed. However, such free energy calculations are still useful in characterizing the differences in thermodynamics and kinetics between these two forms of gp120.

## Discussion

The prerequisite for investigating the dynamics, thermodynamics and kinetics of HIV-1 gp120 is to obtain the full-length structures in both the CD4-bound and CD4-unbound states. Therefore, an attempt has been made to build the as complete as possible gp120 models through a strategy of combining currently available crystal structures of gp120 cores and variable loops as the structural templates. The high sequence identities between the target sequence and templates guarantee the reliability of the constructed structural models. Furthermore, the results of structural quality evaluation by PROCHECK show that for both the models, more than 99% of gp120 residues fall within the favored/allowed regions of the Ramachandran plots (Figure S2). As a result, our two gp120 structural models are suitable for exploring the dynamic and thermodynamic properties of gp120.

The static and dynamic structural/geometric comparisons, together with the results ED analyses, point to a common conclusion that the bound gp120 assumes a more stable, compact conformation than the unbound form. The more compact packing of the bound gp120 can be attributed to its more number of inter-atomic contacts/interactions, which results in more number of longer regular secondary structures and less number of shorter loops/linkers compared to the unbound gp120. The nature to increase the conformational entropy of the protein makes the less-well-restrained loops/linkers fluctuate more intensively than the well-restrained secondary structural elements [29], [30]. In addition, the solvent-exposed loops/linkers are easier to be affected by the mobility of water molecules [35], [36]. Consequently, the unbound gp120, due to its more number of longer loops/linkers, displayed a higher flexibility, a more loose packing, and larger structural deviations compared to the bound gp120 during simulations.

The FELs and free energy profiles constructed based on the metadynamics simulations reveal that the unbound gp120 has a larger, more rugged and complex free energy surface than the bound gp120, thus determining that the unbound gp120 has more conformational substates, a richer conformational diversity, and more complex dynamic behaviors than the bound gp120. According to the FEL theory, the nature to increase the conformational entropy of the protein (especially that of the solvent-exposed loops) and, the competitive interaction between residue-residue and residue-solvent, will inevitable bring out fluctuations of the free energy of the protein-solvent system, which manifest as the rugged characteristics of the free energy surface [28], [29], [36]. For proteins with the same size, the flexible protein generally has more solvent-exposed loops and less regular SSEs than the rigid one. Therefore, the free energy surface of the flexible protein can be expected to be more rugged and complex than that of the rigid protein. As discussed above, the unbound gp120 has a higher flexibility than the bound gp120; this explains why the FEL of the unbound gp120 is more rugged and complex than that of the bound gp120. Like other proteins [62], [63], the more conformational substates of the unbound gp120 caused by its rugged free energy surface may be advantageous in not only recognizing/interacting with multiple structurally dissimilar partners but also in finding out an outlet for transition to the bound state.

The calculated free energy difference between the unbound and bound states of gp120 is ∼−6.0 kJ/mol. The lower free energy of the bound state can be attributed to the formation of the bridging sheet and the presence of more number of inter-atomic interactions (e.g., hydrogen bonds and VDW contacts) compared to the unbound state. Furthermore, such a relatively large free energy gradient will allow a spontaneous transition from the unbound state to the bound state, even if relatively large energy barriers exist between these two states. Therefore, the free energy calculations support our previous speculation (for details, see the Introduction section), and thus explain why the unbound state is hard to be “trapped” in the crystallization condition and why the CD4-bound state rather than the “true” unbound conformation was resolved in the crystallographic study by Kwon et al [21]. In addition, for the isolated gp120, such a spontaneous transition from the unbound to bound states in solution may support the conformational selection mechanism of protein-ligand binding [64], [65], [66]. It should keep in mind that in the functional viral spike of HIV-1, gp120 exists in the gp120_3_/gp41_3_ trimeric assembly, and therefore the interactions of gp120 with gp41 and other subunits are able to maintain its conformation in the unbound state and prevent the spontaneous transition to the bound state. Since the CD4-bound conformation is a stable, free energy minimum state *in vivo*, there are many types of antibodies that recognize this state [21]. The maintenance of the unbound state by the viral spike avoids the transition to the neutralization-sensitive bound state, thus providing an advantage for the viral immune evasion. In the functional viral spike, there must exist a conformational control mechanism responsible for the transition from the unbound to the bound states, and this may be realized by the variable interactions of gp120 with gp41 and by the conformational changes of the surface-exposed large loops of gp120. Taken together, our comparative analysis of the FELs provides a dynamics interpretation of how gp120 molecule escapes antibody neutralization while keeping CD4-binding capacity.

Although in this study only the unbound and bound states of the monomeric gp120 were subjected to MD simulations, the large concerted motions derived from ED analyses can still shed light on the mechanism of conformational interconversion of these two states as well as on the function properties of gp120. The reasons for this are: i) the large concerted motions, despite along the first few eigenvectors, are the equilibrium fluctuations that contribute substantially to the total fluctuation in the conformational space [30]; ii) the fluctuations that can cause conformational transition/denaturation are to a large extent rooted in the equilibrium fluctuations [62]; iii) the biological function of a protein must be governed by the equilibrium fluctuations due to their characteristics of fluctuant stability and persistence [28], [34].

For the unbound and bound gp120, several common features emerge from their large concerted motions: i) the major parts of the inner and outer domains that form the structural core of gp120 can rotate either in the same direction, displaying as a common, large vortex, or in the opposite directions, resulting in two twisting vortices; ii) the structural regions located at the periphery of the vortex have a relatively larger displacement magnitude than the inner of the vortex; iii) the largest structural displacements generally occur in the excursion regions (N-, C-termini and external loops) far from the structural core, including the N- and C-termini, loops V3 and V4, and V1/V2 stem or bridging sheet, which can move either in concert with the large vortex or in the same/opposite directions with respect to one another. Despite the dominant vortical motion modes, the difference in motion direction between the external regions of these two forms of gp120 could lead to different consequences for the conformation of gp120. For instance in the first and second ranked modes of the unbound gp120 (Figures 5A and B), the mutual approach between the V1/V2 stem and V3 loop may benefit the formation of the bridging sheet and as thus facilitates conformational transition to the bound state. In the third and fourth modes of the unbound gp120, the V1/V2 stem moves slightly away from the V3 base, thus preventing the bridging sheet formation and tending to maintain the unbound state. In the first and second ranked modes of the bound gp120 (Figures 5E and F), the two bridging sheet elements move apart from each other, thus tending to disrupt the bridging sheet and facilitating transition to the unbound state. On the contrary, the twisting motion of the bridging sheet observed in the third ranked mode (Figure 5G) seems to stabilize this minidomain, thus facilitating the maintenance of the bound state.

The other consequence of the large concerted motions is that they can affect the size and/or shape of the channel in the unbound gp120 and of the CD4-binding cavity in the bound gp120. For instance for the unbound gp120, its first to fourth ranked modes will lead to the twisting, bending, narrowing, and widening of the long, narrow channel, respectively. For the bound gp120, its first to fourth ranked modes will lead to the bending, twisting, widening, and narrowing of the CD4-binding cavity, respectively. These modes could be related to the functional properties of gp120 such as the recognition, binding, orientation, and release of the ligands as well as the regulation of these processes.

The largest displacement magnitude of the excursion regions originates from their weakest structural restraints and direct contacts with the solvent water. Such large displacements, despite being far away from the gp120 core, can influence the structural fluctuations of the core periphery and further, transmit over the entire structure via specific mechanic mechanism (e.g. the hinge mechanism) and water network around the protein surface, thus causing the large concerted motions relevant to the functional properties and conformational transition. Visualization of the 2D essential subspaces explored by the different structural components of both the unbound and bound gp120 (Figure S3) reveals that i) the excursions (N-, C-termini and external loops) explore the largest regions in conformational space while the core periphery that participates in interactions with the external loops and the core inner that has no direction contact with the external loops explore the moderate and the smallest regions, respectively; ii) different structural components, especially the external loops, core periphery, and core inner, sample similar regions (or similar shapes of the sampled regions) in conformational space. To this end, it is reasonable to believe that the excursions play a crucial role in influencing or even determining the dynamics of gp120. It can be expected that in the context of the functional viral spike, the excursions could also play a role in influencing and regulating the dynamics of gp120 via their variable interactions with gp41 and other structural components from the adjacent subunits of the gp120_3_/gp41_3_ trimeric complex. In addition, the large displacements of the external loops are able to cause multiple conformational substates, particularly in the case of the unbound gp120 as discussed above, which provide an advantage for the immune evasion of HIV-1 [21]. Interestingly, the large displacements of the V3 loop in the bound gp120 may facilitate initial recognition and binding of the co-receptor due to the fact that it is a crucial structural component involving interaction with the co-receptor [67], [68].

The combined ED analysis reveals that the first eigenvector separates well the large concerted motions between the unbound and bound forms of gp120 (Figures 6A, B, and C). Although such motions are only along the first combined eigenvector, they are the most significant equilibrium fluctuations within the essential subspace [30]. Therefore, such a significant difference could be understood as a large difference in equilibrium/average conformational states between the unbound and bound gp120, supporting the results of free energy calculations. Along the combined eigenvectors 2–4, the gradually increasing overlaps between the projection distributions reveal a gradually increasing similarity between the essential degrees of freedom of the unbound and bound gp120. This may be considered as one of the dominant reasons for interconversion of these two states. The combined ED analysis also reveals significantly larger conformational shifts of the unbound gp120 than the bound gp120 along the first five eigenvectors, and this will lead to a richer conformational diversity and more substates of the unbound than the bound gp120, in agreement with the analysis of FELs.

## Conclusions

We have built the near-full-length gp120 structural models in the unbound and bound states and investigated the dynamics, molecular motions and FEL characteristics of these two forms of gp120 based on the standard MD and metadynamics simulations. The comparisons in the static structures, dynamic geometric properties, RMSF values, and ED properties between these two forms of gp120 commonly reveal that the bound gp120 assumes a more compactly packed and stable conformation than the unbound gp120. The more compact packing of the bound gp120 can be attributed to its more number of inter-atomic contacts/interactions, which in turn result in stronger structural constraints and hence more stable fluctuations of the bound gp120 than the unbound gp120 during simulations. The free energy calculations show that the FEL of the unbound gp120 is more rugged and complex than that of the bound gp120. This determines that gp120 has more conformational substates, a richer conformational diversity, and more complex dynamic behaviors in the unbound state than in the bound state. The estimated difference in the absolute minimum free energy between the unbound and bound gp120 is ∼−6.0 kJ/mol, thus allowing the spontaneous transition from the unbound state to the bound state by following such a relatively large energetic gradient. The large-scale motions along the first few eigenvectors can be described as large rotational vortices formed by the concerted movements of the major parts of the inner and outer domains. The excursions (i.e., the N- and C-termini, loops V3 and V4, and V1/V2 stem or bridging sheet) can move either in concert with the large vortices or in the same/opposite directions with respect to one another. Because these motion modes have effects on the size/shape of the ligand-binding channel/cavity of gp120, they were proposed to be related to the functional properties of gp120. In addition, the variance in motion directions of certain structural components, and the gradually increasing overlap between the projection distributions along the first few combined eigenvectors of the unbound and bound gp120, indicate that the large concerted motions govern the conformational interconversion of the unbound and bound states. Finally, we highlight that the large displacements of the external loops and the N- and C-termini are crucial in influencing and regulating the dynamics, thermodynamics and kinetics of gp120. Our results will facilitate understandings of the gp120 functional properties, the mechanism of gp120 conformational control, and as a result the immune-evasion mechanism of HIV-1.

## Supporting Information

Figure S1 **Time evolution of the backbone RMSD values of the unbound gp120 (A) and the bound gp120 (B) with respect to their respective starting structures during the 6 independent MD simulations (replicas 1–6).**
(TIF)

Figure S2 **Ramachandran plots of the constructed near-full-length gp120 models.** (A) Unbound gp120. (B) Bound gp120.
(TIF)

Figure S3 **Projections of the simulation trajectories for the different structural components onto the first two eigenvectors.** These projections represent the 2D essential subspaces explored by the N- and C-termini (A), external loops (B), core periphery that participates in interactions with the external loops (C), and core inner that has no direct contact with the external loops (D) of the unbound gp120. (E)–(H) represent the essential subspaces explored by the above corresponding structural components of the bound gp120, respectively.
(TIF)

Table S1 **Cosine content values of the first 4 eigenvectors calculated from the 6 independent equilibrium MD trajectories (5–15 ns; replicas 1–6) and the single 60-ns joined trajectories.**
(DOCX)

Table S2 **Average backbone RMSD values and corresponding standard deviations (in parentheses) for the near-full-length gp120 models and the gp120 cores with respect to their respective starting structures calculated from the equilibrium trajectories (5–15 ns) of the 6 independent MD simulations (replicas 1–6).**
(DOCX)
