# Supplementary figures and images for: Insight Derived from Molecular Dynamics Simulations into Molecular Motions, Thermodynamics and Kinetics of HIV-1 gp120
Source: PLoS One. 2014 Aug 8;9(8):e104714. doi: 10.1371/journal.pone.0104714 (PMC4126740; doi:10.1371/journal.pone.0104714)

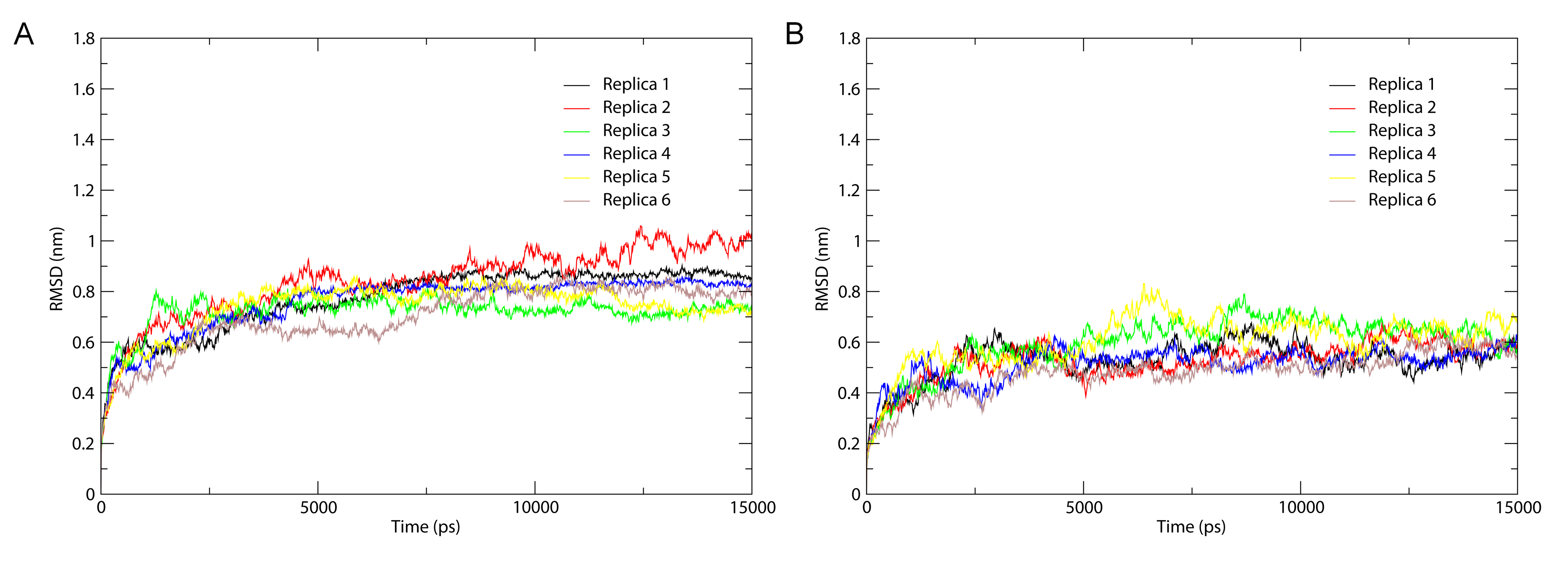

Supplement: Figure S1 — Time evolution of the backbone RMSD values of the unbound gp120 (A) and the bound gp120 (B) with respect to their respective starting structures during the 6 independent MD simulations (replicas 1–6). (TIF) [file pone.0104714.s001.tif]

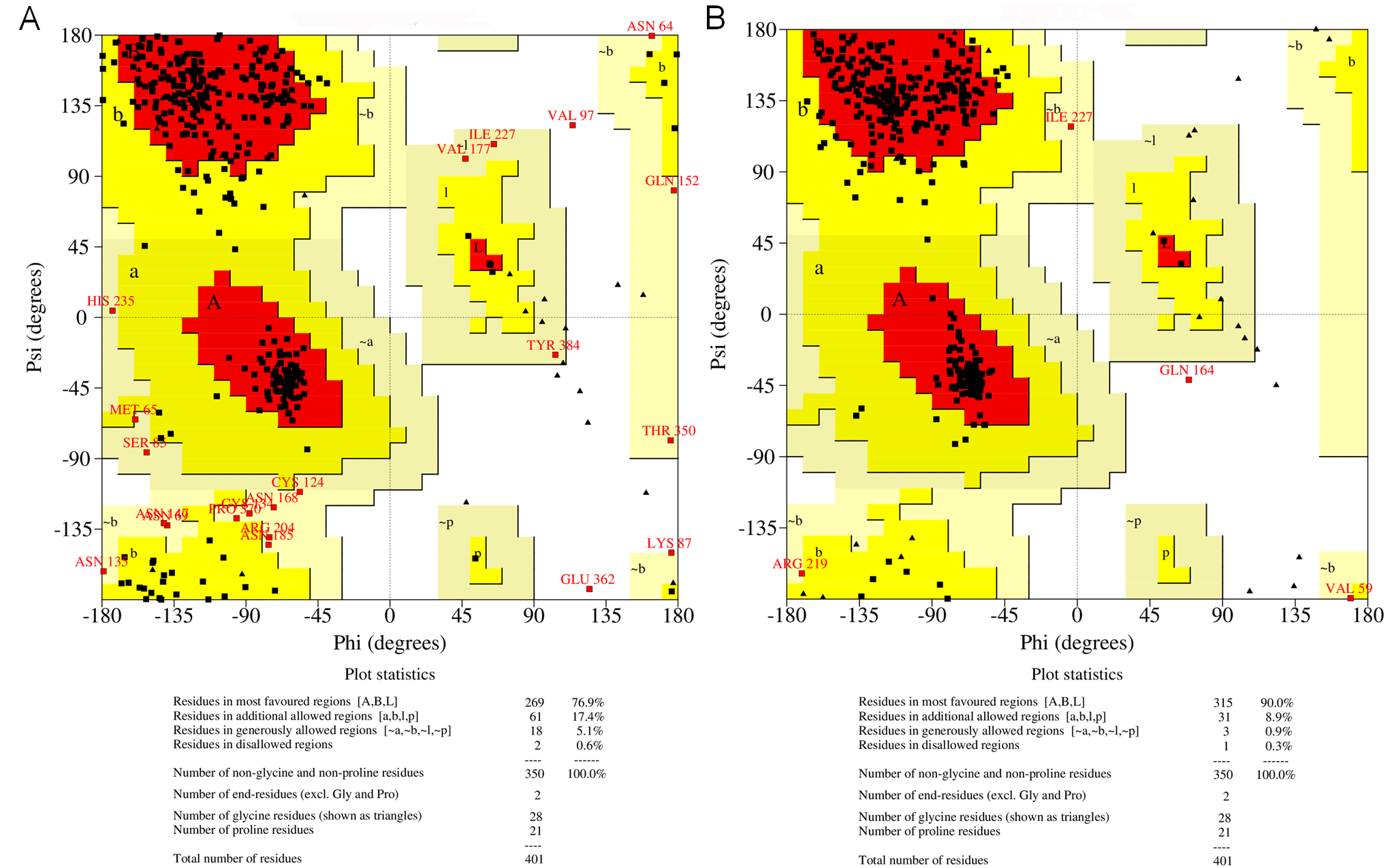

Supplement: Figure S2 — Ramachandran plots of the constructed near-full-length gp120 models. (A) Unbound gp120. (B) Bound gp120. (TIF) [file pone.0104714.s002.tif]

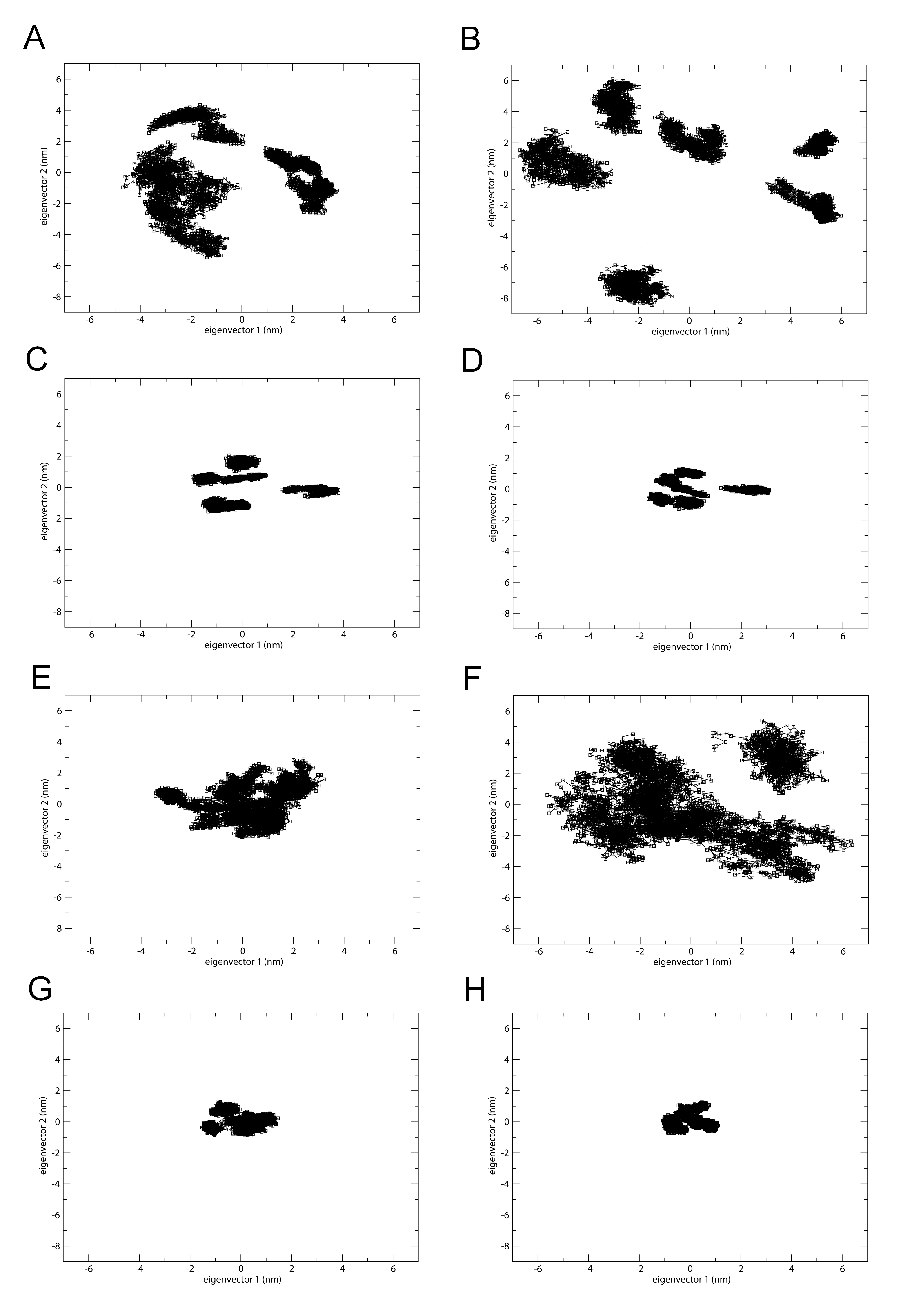

Supplement: Figure S3 — Projections of the simulation trajectories for the different structural components onto the first two eigenvectors. These projections represent the 2D essential subspaces explored by the N- and C-termini (A), external loops (B), core periphery that participates in interactions with the external loops (C), and core inner that has no direct contact with the external loops (D) of the unbound gp120. (E)–(H) represent the essential subspaces explored by the above corresponding structural components of the bound gp120, respectively. (TIF) [file pone.0104714.s003.tif]
