# Supplementary material for: Insight Derived from Molecular Dynamics Simulations into Molecular Motions, Thermodynamics and Kinetics of HIV-1 gp120
Source: PLoS One. 2014 Aug 8;9(8):e104714. doi: 10.1371/journal.pone.0104714 (PMC4126740; doi:10.1371/journal.pone.0104714)
Supplement: Table S1 — Cosine content values of the first 4 eigenvectors calculated from the 6 independent equilibrium MD trajectories (5–15 ns; replicas 1–6) and the single 60-ns joined trajectories. (DOCX) [file pone.0104714.s004.docx]

Table S1. Cosine content values of the first 4 eigenvectors calculated from the 6 independent equilibrium MD trajectories (5-15 ns; replicas 1-6) and the single 60-ns joined trajectories.

| traj. | unbound gp120 | | | | bound gp120 | | | |
| --- | --- | --- | --- | --- | --- | --- | --- | --- |
|  |  |  |  |  |  |  |  |  |
|  | eig.1 | eig.2 | eig.3 | eig.4 | eig.1 | eig.2 | eig.3 | eig.4 |
| 1 | 0.7166 | 0.2504 | 0.2064 | 0.2707 | 0.0137 | 0.023 | 0.0753 | 0.0877 |
| 2 | 0.7734 | 0.4444 | 0.3465 | 0.0158 | 0.8768 | 0.3217 | 0.0393 | 0.0173 |
| 3 | 0.9240 | 0.1206 | 0.0013 | 0.1892 | 0.8540 | 0.6031 | 0.0445 | 0.0002 |
| 4 | 0.9212 | 0.7798 | 0.6247 | 0.1540 | 0.7447 | 0.6930 | 0.1356 | 0.0592 |
| 5 | 0.9236 | 0.7942 | 0.0014 | 0.0640 | 0.0804 | 0.0252 | 0.0655 | 0.0569 |
| 6 | 0.6890 | 0.2722 | 0.0866 | 0.0001 | 0.8799 | 0.6881 | 0.0859 | 0.0984 |
| joined | 0.0280 | 0.0800 | 0.0586 | 0.0089 | 0.0024 | 0.0072 | 0.0471 | 0.0005 |
