# Supplementary material for: Insight Derived from Molecular Dynamics Simulations into Molecular Motions, Thermodynamics and Kinetics of HIV-1 gp120
Source: PLoS One. 2014 Aug 8;9(8):e104714. doi: 10.1371/journal.pone.0104714 (PMC4126740; doi:10.1371/journal.pone.0104714)
Supplement: Table S2 — Average backbone RMSD values and corresponding standard deviations (in parentheses) for the near-full-length gp120 models and the gp120 cores with respect to their respective starting structures calculated from the equilibrium trajectories (5–15 ns) of the 6 independent MD simulations (replicas 1–6). (DOCX) [file pone.0104714.s005.docx]

Table S2. Average backbone RMSD values and corresponding standard deviations (in parentheses) for the near-full-length gp120 models and the gp120 cores with respect to their respective starting structures calculated from the equilibrium trajectories (5-15 ns) of the 6 independent MD simulations (replicas 1-6).

| replica | RMSD (nm) | | | |
| --- | --- | --- | --- | --- |
|  | unbound | unbound_core | bound | bound_core |
| 1 | 0.77(0.12) | 0.57(0.06) | 0.53(0.08) | 0.31(0.03) |
| 2 | 0.84(0.14) | 0.65(0.07) | 0.53(0.08) | 0.32(0.03) |
| 3 | 0.72(0.07) | 0.59(0.06) | 0.60(0.11) | 0.38(0.06) |
| 4 | 0.76(0.11) | 0.57(0.06) | 0.52(0.07) | 0.29(0.03) |
| 5 | 0.74(0.10) | 0.56(0.06) | 0.61(0.10) | 0.36(0.05) |
| 6 | 0.70(0.12) | 0.52(0.07) | 0.48(0.08) | 0.32(0.05) |
